# Src mediates cytokine-stimulated gene expression in airway myocytes through ERK MAPK

**DOI:** 10.1186/1478-811X-9-14

**Published:** 2011-05-20

**Authors:** Cherie A Singer, Beata Lontay, Helmut Unruh, Andrew J Halayko, William T Gerthoffer

**Affiliations:** 1 University of Nevada School of Medicine, Department of Pharmacology Reno, NV 89557, USA; 2 University of Manitoba, Section of Thoracic Surgery, Winnipeg, MB, Canada R3A 1R9; 3 University of Manitoba, Department of Physiology and Section of Respiratory Diseases, Winnipeg, MB, Canada R3A 1R8; 4 University of South Alabama, Department of Biochemistry & Molecular Biology, Mobile, AL 36688, USA

## Abstract

The p38 and extracellular signal-regulated kinases (ERK) mitogen-activated protein kinases (MAPK) participate in cytokine-stimulated inflammatory gene expression in airway smooth muscle cells. The following study was undertaken to determine whether Src tyrosine kinases are signaling intermediaries upstream of cytokine-stimulated MAPK activation and gene expression. Treating human airway myocytes with interleukin (IL)-1β, tumor necrosis factor (TNF) α and interferon (IFN) γ caused a rapid 1.8-fold increase in Src family tyrosine kinase activity within 1 minute that remained 2.3 to 2.7 fold above basal conditions for 15 minutes. This activity was blocked by addition of 30 μM PP1, a pyrimidine inhibitor specific for Src family tyrosine kinases, in immune-complex assays to confirm that this stimulus activates Src tyrosine kinase. Addition of PP1 also blocked cytokine-stimulated expression of IL-1β, IL-6 and IL-8, while decreasing phosphorylation of ERK, but not p38 MAPK. Since this inflammatory stimulus may activate additional inflammatory signaling pathways downstream of Src, we tested the effects of PP1 on phosphorylation of signal transducers and activators of transcription (STAT). PP1 had no effect on cytokine-stimulated STAT 1 or STAT 3 phosphorylation. These results demonstrate that Src tyrosine kinases participate in the regulation of IL-1β, IL-6 and IL-8 expression and that these effects of Src are mediated through activation of ERK MAPK and not p38 MAPK or STAT1/STAT3 phosphorylation.

## Findings

Our laboratory has examined signaling pathways regulating secretion of inflammatory mediators by human airway smooth muscle cells. The synthesis and secretion of Th1/Th2 cytokines, along with CC and C-X-C chemokines, chemotactic proteins, peptide growth factors and their receptors can be induced in these myocytes by exposure to, among others, interleukin (IL)-1β, tumor necrosis factor (TNF) α, interferon (IFN) γ, or transforming growth factor β [1,2] and contributes to inflammatory airway disease. In previous studies, we used a complementary DNA expression array to analyze expression of inflammatory mediators following treatment with a pro-inflammatory stimulus consisting of IL-1β, TNFα and IFNγ and established that this stimulus induces expression of multiple inflammatory mediators including IL-1β, IL-6, and IL-8 [3]. Pharmacological inhibitors of mitogen-activated protein kinase (MAPK) activation were used to further demonstrate that both p38 and ERK MAPK are upstream mediators of IL-6 and IL-8 expression, while ERK MAPK alone was involved in mediating IL-1β expression.

Src tyrosine kinases are one of the signaling intermediaries linking multiple types of receptors to MAPK activation in smooth muscle. In colonic myocytes, the G-protein coupled M_2 _muscarinic receptor is coupled to ERK MAPK through Src activation [4] and expression of IL-1β, IL-6, IL-8, and cyclooxygenase (COX-2) mRNA is reduced by inhibition of Src with the pyrimidine inhibitor PP1 [5]. In vascular smooth muscle cells, angiotensin II stimulates Src-dependent p38 MAPK activation [6] and CD40 ligation initiates Src activation of p38 and ERK MAPK, resulting in the induction of IL-8 and monocyte chemotactic protein-1 [7]. In airway myocytes, the PDGF receptor signals to ERK MAPK through Src activation via a pertussis-toxin sensitive mechanism that suggests the involvement of G_i_-protein subunits [8] and sphingosine-1-phosphate stimulation of a G_i_-coupled receptor also stimulates ERK MAPK through Src [9]. In addition to acting as an upstream mediator of MAPK, Src may also activate other signaling pathways that could affect inflammatory gene expression, such as the Janus kinase-signal transducers and activators of transcription (JAK-STAT). This is supported by evidence that inhibition of Src in PDGF-stimulated airway myocytes with PP2 blocks both ERK MAPK and JAK2 activation, resulting in decreased phosphorylation of STAT1 and STAT3 [10] that requires an interaction with the small GTPase Rac1 [11].

**Figure 1:**
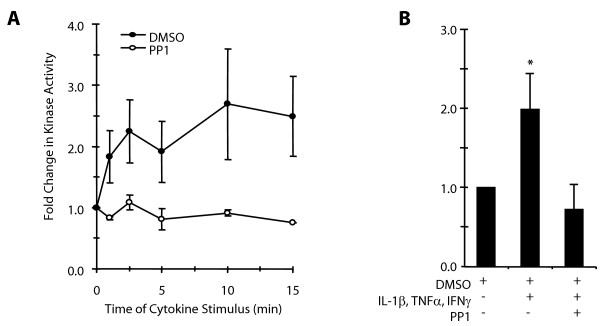
**Effect of cytokine stimulation on Src tyrosine kinase activity in airway myocytes**. **A**. Cultures were treated at the times indicated with 10 ng/ml IL-1β, TNFα and IFNγ in the presence of the 0.1% DMSO vehicle *(closed circles) *or 30 μM PP1 *(open circles)*. *In vitro *Src kinase assays were performed using the synthetic peptide p34^cdc2 (6-20) ^as the substrate, n = 3 ± SEM. **B**. Cultures were treated for 5 minutes with 10 ng/ml IL-1β, TNFα and IFNγ in the presence of the 0.1% DMSO vehicle or 30 μM PP1. Immune-complex kinase assays were performed as above in Src immunoprecipitates. Data are expressed as the fold change relative to the absence of stimulus, n = 4 ± SEM. * indicates significant difference from unstimulated or PP1 treated cultures, p < 0.05.

The following study was undertaken to determine whether activation of Src is an event upstream of MAPK signaling contributing to cytokine-stimulated gene expression and all methodological details are described in Additional File 1. *In vitro *kinase assays initially evaluated the activity of Src in human airway myocytes isolated according to previously published protocols using p34^cdc2 (6-20) ^as the substrate. This substrate is specific for Src tyrosine kinase family members including Src, Lck, Lyn and Fyn but not non-Src tyrosine kinases such as Abl [12]. Stimulation of cultures with 10 ng/ml IL-1β, TNFα and IFNγ caused a rapid 1.8-fold increase in Src family tyrosine kinase activity within 1 minute that remained 2.3 to 2.7-fold above basal conditions for 15 minutes (Figure 1A) and addition of the inhibitor PP1 prevented cytokine stimulation of kinase activity. This inhibitor, and the similar compound PP2, have been widely used to pharmacologically inhibit multiple Src family members [13-15], and PP1 can also inhibit Abl, which was presumably not measured in our assay with the p34^cdc2 (6-20) ^substrate. To confirm the activity measured was attributed to Src, we performed additional immune-complex kinase assays using the p34^cdc2 (6-20) ^substrate following immunoprecipitation with a Src-specific antibody, as described [4]. In these assays (Figure 1B), a 2-fold increase in activity of Src immunoprecipitates within 5 minutes of stimulation was abolished by addition of 30 μM PP1, indicative of Src-specific activity in airway myocytes.

**Figure 2:**
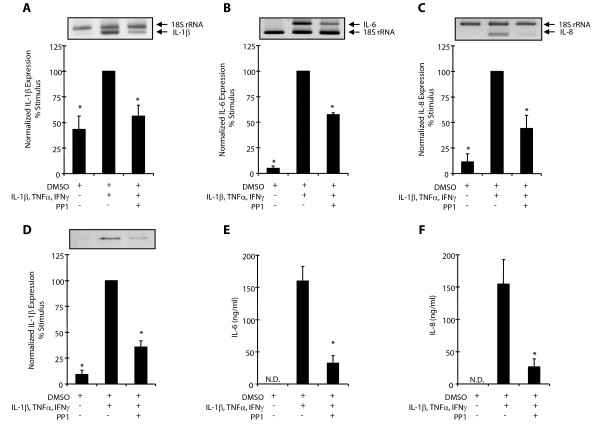
**PP1 inhibits cytokine expression and synthesis**. Human airway myocytes were treated for 20 hours with 10 ng/ml IL-1β, TNFα and IFNγ in the presence of the 0.1% DMSO vehicle + 30 μM PP1. **A-C**. Relative multi-plex RT-PCR amplifying products of the predicted size for 18S rRNA (324 bp), IL-1β (291 bp), IL-6 (425 bp), and IL-8 (188 bp) with a graphical summary of expression normalized to 18S rRNA, n = 4 + SEM. Data is expressed as the % change from cytokine stimulated cultures (100%). **D**. Western analysis demonstrating intracellular pro-IL-1β immunoreactivity at 33 kDa, n = 5 + SEM. **E-F**. IL-6 or IL-8 secretion measured by ELISA, n = 3 + SEM; N.D. = not detected; * indicates significant difference from cytokine stimulated cultures, p < 0.05.

To determine whether Src activation contributes to inflammatory gene expression, we chose to evaluate the expression of genes previously shown to be regulated by p38 and ERK MAPK, namely IL-1β, IL-6 and IL-8 [3]. Gene expression was measured using a relative semi-quantitative RT-PCR approach in which the target gene is amplified within the linear range in a multi-plex reaction containing 18S rRNA as an endogenous standard. We further verified the results of these experiments by quantifying synthesis of the target protein by Western blots or ELISA. As shown previously [3], expression of IL-1β, IL-6 and IL-8 is induced upon stimulation with 10 ng/ml IL-1β, TNFα and IFNα for 20 hours (Figure 2). Treatment with 30 μM PP1 reduces cytokine-stimulated IL-1β mRNA expression by 53% (Figure 2A), which correlates by a 64% decrease in IL-1β synthesis, as measured by immunoblotting for the intracellular 33 kDa form of IL-1β (Figure 2D). This was necessary since high levels of IL-1β present in the media from the cytokine-stimulus would hamper results from ELISA. Treatment with PP1 also inhibited IL-6 mRNA expression by 43% (Figure 2B) and IL-8 mRNA expression by 56% (Figure 2C), which again corresponded to a 80% decrease in IL-6 secretion from 160.8 ng/ml to 32.9 ng/ml (Figure 2E) and a 82% decrease in IL-8 secretion from 155.6 ng/ml to 27.2 ng/ml (Figure 2F). Thus, the same concentration of PP1 that effectively blocks Src kinase activity results in inhibition, but not complete blockade, of inflammatory gene expression. This suggests that while Src activity is involved in expression of the genes examined, additional Src-independent signaling pathways likely participate.

**Figure 3:**
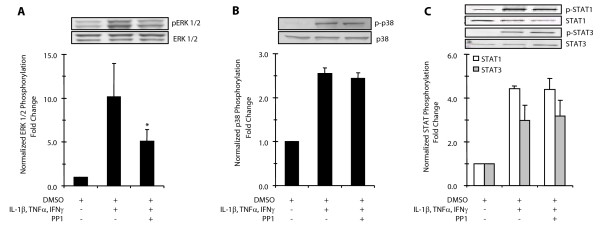
**Effects of PP1 on cytokine-stimulated MAPK phosphorylation**. Human airway myocytes were treated for 15 minutes with 10 ng/ml IL-1β, TNFα and IFNγ in the presence of the 0.1% DMSO vehicle + 30 μM PP1. Aliquots of whole cell lysates were separated by SDS-PAGE and immunoblots performed to examine phosphorylation of ERK (5 μg total protein) and p38 MAPKs (15 μg total protein). **A**. Effect of PP1 on cytokine-stimulated ERK 1/2 MAPK phosphorylation (pERK 1/2) normalized to non-phosphorylated ERK. **B**. Effect of PP1 on cytokine-stimulated p38 MAPK phosphorylation (p-p38) normalized to non-phosphorylated p38 MAPK. Representative immunoblots are shown along with normalized densitometric data expressed relative to the immunoreactivity seen in DMSO treated control cultures, n = 4 + SEM. * indicates significant difference from cytokine-stimulated group, P < 0.05.

To determine whether Src is an upstream mediator of MAPK activation in airway myocytes, we analyzed MAPK activation by phospho-immunoblot analysis in cultures treated with PP1. Treatment with 10 ng/ml IL-1β, TNFα and IFNγ for 15 minutes resulted in a 10.2 fold increase in ERK MAPK phosphorylation that was inhibited 50% by addition of PP1 (Figure 3A). The same stimulus resulted in a 2.5 fold increase in p38 MAPK phosphorylation but addition of PP1 had no effect (Figure 3B). Since previous reports demonstrated that PDGF stimulation of airway myocytes results in Src-dependent phosphorylation of STAT1/STAT3 via JAK2 activation [10], we determined whether the inflammatory stimulus used here may activate JAK-STAT signaling pathways to mediate downstream gene expression (Figure 3C). The combination of 10 ng/ml IL-1β, TNFα and IFNγ increased STAT1 phosphorylation 2.5 fold and STAT3 phosphorylation 3.7 fold above basal levels within 10 minutes. However, addition of 30 μM PP1 did not affect STAT phosphorylation, indicating that this with this inflammatory stimulus, Src does not mediate JAK-STAT signaling.

Clearly, the functions of Src tyrosine kinases in smooth muscle cells are varied and likely involve interactions with multiple signaling pathways. In the airway, Src mediates serotonin-evoked peak Ca^2+ ^responses by affecting phosphoinositide levels to alter cell contraction [15]. Src also mediates PDGF and thrombin-induced proliferative responses, possibly through stimulation of cyclin D1 expression [10,13]. While previous studies have established that both ERK and p38 MAPK are involved in regulating expression of many inflammatory genes in airway myocytes [3,16,17], this work demonstrates that Src tyrosine kinases also play a role in regulating inflammatory gene expression by signaling upstream of ERK but not p38 MAPK. This is supported by studies of inflammatory gene expression in other cell types. In pulmonary epithelial cells, both ERK and p38 MAPK contribute to silica-induced IL-8 release but only ERK MAPK activation is dependent on Src [14]. CD40 stimulation of IL-8 and MCP-1 production in vascular myocytes is also dependent on ERK and p38 MAPK activation but the inhibitor PP2 was found to inhibit only ERK MAPK [7]. In this same study, PP2 also decreased activation of IκB kinase, indicating that Src stimulates NF-κB signaling to affect chemokine production and suggesting that NF-κB activation could link cytokine stimulation to MAPK activation. We have previously shown that IL-1β and TNFα but not IFNγ activates NF-κB in airway myocytes and inhibition of NF-κB activity reduces expression of IL-1β, IL-6, IL-8 and COX-2 in a manner independent of p38 MAPK [5,16].

The link between inflammatory signaling and Src-dependent MAPK activation in our studies remains to be determined. One possibility may be the TNF receptor-associated proteins (TRAFs). Binding of IL-1β to the IL-1 receptor activates IL-1 receptor associated kinases that recruit TRAFs. TNF receptors also recruit TRAFs through the TNF receptor DEATH domain (TRADD). In fibroblasts, Src interacting with TRAF2 links TNFα stimulation to ERK MAPK [18] and TRAF proteins also activate NF-κB through recruitment of IκB kinase (see review by [19]). The contribution of IFNγ signaling to Src-dependent ERK MAPK activation is less clear. Interactions between Src tyrosine kinases and receptor-associated JAKs are often required for complete STAT activation [20] but downstream activation of ERK MAPK has not been demonstrated. It has been proposed that IFNγ can directly activate ERK MAPK pathways through MEKK1 [21] but the signaling intermediates, if any, are unknown. Another possibility could involve heterotrimeric G_i _subunits implicated in Src-dependent activation of ERK MAPK in airway myocytes stimulated with PDGF [8]. The contribution of these proteins have not been widely examined in the context of IL-1β TNFα or IFNγ signaling, which are more likely to utilize small GTPases such as Rac1 [11] and RhoA [22]. Thus, while these results demonstrate that Src participate in the regulation of inflammatory gene expression through activation of ERK MAPK, further studies will explore the signaling pathways linking Src-dependent cytokine stimulation to MAPK activation.

## Competing interests

The authors declare that they have no competing interests.

## Authors' contributions

CAS and BL performed all the experiments. HU and AJH provided and characterized human cells used in the experiments. CAS wrote the manuscript and AJH and WTG provided comments for revision. All authors have read and approved the final manuscript.

## Supplementary Material

Additional file 1 **The file provided detailed methods described in the communication **[23]. Click here for file
