# Supplementary material for: Src mediates cytokine-stimulated gene expression in airway myocytes through ERK MAPK
Source: Cell Commun Signal. 2011 May 20;9:14. doi: 10.1186/1478-811X-9-14 (PMC3123314; doi:10.1186/1478-811X-9-14)
Supplement: Additional file 1 — The file provided detailed methods described in the communication [23]. [file 1478-811X-9-14-S1.DOC]

**Materials and Methods**

***Cell Culture and Drug Treatments***

Primary human airway myocytes were obtained from 2^nd^-4^th^ generation mainstem bronchi of patients undergoing lung resection surgery in accordance with procedures approved by the Human Research Ethics Board of the University of Manitoba, Winnipeg, Canada, as previously described [23]. Myocytes were maintained in a humidified 5% CO_2_ atmosphere at 37°C in M199 supplemented with 5% fetal bovine serum (FBS), 0.5 ng/ml epidermal growth factor, 5 μg/ml insulin, 2 ng/ml fibroblast growth factor, 50 μg/ml gentamicin and 50 ng/ml amphotericin B. Cultures from passages four through eight were used for these experiments. When the cultures reached confluence, they were growth arrested for 24 hours in media supplemented with 0.1% FBS, growth factors, antibiotics and ITS+ (6.25 μg/ml insulin, 6.25 μg/ml transferrin, 6.25 ng/ml selenious acid and 5.35 μg/ml linoleic acid) prior to further treatments.

***Src Tyrosine Kinase Assays***

Cultures were pre-incubated for 15 minutes with a 0.1 % DMSO vehicle control or 30 μM of the Src inhibitor PP1 and stimulated with 10 ng/ml IL-1β, TNFα and IFNγ at the times indicated. Whole cell lysates were prepared from by homogenization and centrifugation in 50 mM HEPES, 150 mM NaCl, 1 mM sodium orthovanadate, 10 mM NaF, 0.1 mM AEBSF, 1 μM leupeptin, 0.5 % Triton X-100, 0.5 % NP-40 and 10% glycerol. Protein concentrations were determined using the bicinchoninic acid method using bovine serum albumin as the standard. Twenty μg of total protein was incubated for 60 min at 30°C in 4X kinase assay buffer [100 mM Tris-HCl, pH 7.2, 125 mM MgCl2, 25 mM MnCl2, 2 mM EGTA, 0.25 mM sodium orthovanadate and 2 mM dithiothreitol], 10 μCi [γ-^32^P] ATP and 150 μM of the synthetic peptide substrate p34^cdc2 (6-20)^, as directed by the manufacturer (Upstate, Lake Placid, NY). Reactions were then spotted onto P81 phosphocellulose and washed in 0.75% H_3_PO_4_ followed by scintillation counting.

For immune-complex assays, cultures were pre-incubated for 15 minutes with a 0.1 % DMSO vehicle control or 30 μM PP1 and stimulated with 10 ng/ml IL-1β, TNFα and IFNγ for 5 minutes. Src was immunoprecipitated from whole cell lysates by incubating 75 μg of total protein for 2 hours with goat anti-Src (c-Src, N-16, Santa Cruz Biotechnologies, Santa Cruz, CA) in the presence of Protein A/G agarose beads and washed as previously described [4]. This antibody is kinase-specific and according to the manufacturer does not cross-react with other known Src tyrosine kinases. Aliquots from the immunoprecipitates were separated by 8% SDS-PAGE and immunoblots performed with a rabbit anti-Src antibody (Src2, Santa Cruz Biotechnologies, Santa Cruz, CA) to monitor immunoprecipitation using alkaline phosphatase conjugated goat anti-rabbit IgG (data not shown). The remaining immunoprecipitates were incubated for 60 min at 30°C in 4X kinase assay buffer, 10 μCi [γ-^32^P] ATP and 150 μM of the synthetic peptide substrate p34^cdc2 (6-20)^, spotted onto P81 phosphocellulose and washed in 0.75% H_3_PO_4_, followed by scintillation counting.

***RNA Isolation and Relative RT-PCR***

Cultures were treated with 0.1% of the DMSO vehicle or 30 μM PP1 for 15 minutes prior to and during a 20 hour treatment with a cytokine cocktail containing 10 ng/ml IL-1β, TNFα, and INFγ. Total RNA was extracted with TRIzol reagent at 1ml/10 cm^2^ according to the manufacturer’s instructions. Following TRIzol extraction, RNA samples were dissolved in nuclease-free water and treated with 5 units DNase I for 10 minutes at 37°C. The reaction was stopped by addition of 25 mM EDTA and incubation at 65°C for 15 minutes. First strand cDNA synthesis was performed at 42°C from 2 μg total RNA using 250 ng random hexamers, 50 mM Tris-HCl pH 8.3, 75 mM KCl, 3 mM MgCl_2_, 10 mM DTT, 0.125 mM dATP, dTTP, dGTP and cCTP, and 1 unit SuperScript II reverse transcriptase. Following cDNA synthesis, 20 units of RNAse H was then added to remove RNA complementary to the cDNA.

Cytokine genes were amplified by semi-quantitative relative RT-PCR using QuantumRNA 18S internal standards (Ambion, Inc., Austin, TX) and oligonucleotide primer sequences purchased from Clontech (Palo Alto, CA) as previously described [3]. All amplification took place within the linear range of amplification for each gene using with an optimized ratio of 18S primers as an endogenous standard along with 18S competimers that allow modulation of 18S amplification without affecting the performance of the gene specific PCR targets. PCR products were quantified by ethidium bromide staining and analyzed by densitometry. Results were normalized to the amount of 18S rRNA present in each sample.

***Immunoblotting***

Whole cell lysates were prepared in extraction buffer containing 20 mM Tris, pH 7.5, 5 mM [ethylene-bis(oxyethylenenitrilo)]­ tetraacetic acid, 150 mM NaCl, 1% NP40, 0.1 mM Na_3_VO_4_, 1mM NaF, 10 mM sodium β-glycerophosphate, 0.1 mM PMSF, 1 μg/ml leupeptin, 1 μg/ml pepstatin A, 10 μg/ml trypsin inhibitor, and 10 μg/ml aprotinin. Cellular extracts were clarified by centrifugation at 10,000 g for 10 min at 4°C, and supernatants were used to assay intracellular IL-1β levels. Protein concentrations were determined by the bicinchoninic acid method using bovine serum albumin as the standard. To assess IL-1β levels, 20 μg of total protein was separated by 12% SDS-PAGE. Proteins were transferred to nitro­cellulose paper in 25 mM Tris, 192 mM glycine, 10% methanol using a Mini-Genie Blotter (Idea Scientific, MN; 24 V for 2 hours, under continuous cooling at 4°C). Blots were blocked with 0.5% gelatin for 1 hour. Intracellular IL-1β blots were probed with an IL-1β primary antibody (1:500, R&D Systems, Minneapolis, MN) and anti-goat-IgG alkaline phosphatase secondary antibody (1:10,000). Images of immunoblots scanned with a UMAX Powerlook flatbed scanner were analyzed by densitometry.

Immunoblotting for ERK MAPK, p38 MAPK, STAT1 or STAT3 proceeded in blots blocked in Odyssey blocking buffer (Licor Biosciences, Lincoln, NE) diluted 1:1 in PBS for 1 hour. ERK MAPK blots were probed with a dual anti-phosphotyrosine-threonine-p42/p44 and a p44 primary antibody that cross-reacts with p42 (1:1000). p38 MAPK blots were probed with a dual anti-phosphotyrosine-threonine-p38 MAPK and p38 MAPK primary antibodies (1:1000). STAT1 or STAT3 were probed with a STAT isoform specific phosphotyrosine (1:1000) and STAT isoform specific primary antibodies (1:500). Secondary antibodies were conjugated to AlexaFluor® 680 (Molecular Probes, Eugene, OR) or IRDye™800 (Rockland Immunochemicals, Gilbertsville, PA) for fluorometric detection using an Odyssey infrared imaging system (Licor Biosciences, Lincoln, NE). All analyses took place within the linear range of the immunoreactive signal. Antibodies recognizing phosphorylated forms of ERK 1/2 and p38 MAPK were obtained from Cell Signaling Technology (Beverly, MA). All other antibodies for MAPKs or STATs were from Santa Cruz Biotechnologies (Santa Cruz, CA).

***ELISA***

IL-6 and IL-8 secretion was measured in the media of treated cultures by ELISA according to the manufacturer’s protocols (R&D Systems, Minneapolis, MN). The minimum detectable levels of IL-6 and IL-8 within the assay were 0.7 pg/ml and 10 pg/ml, respectively.

***Densitometry and Statistical Analysis***

Densitometry for RT-PCR was performed using a BioRad Model 525 Molecular Imager and the Volume Analyze feature of Molecular Analyst software (BioRad, Hercules, CA). Densitometry for immunoblots was performed using the Odyssey infrared imaging system software (Licor Biosciences, Lincoln, NE). Statistical analysis was performed by one-way ANOVA followed by post-hoc testing with the Student-Newman-Keuls method using SigmaStat software (Jandel Scientific, San Rafael, CA).
